# Droplet Flow Assisted Electrocatalytic Oxidation of Selected Alcohols under Ambient Condition

**DOI:** 10.3390/molecules27020382

**Published:** 2022-01-07

**Authors:** Mohammed A. Suliman, Khaled M. Al Aqad, Chanbasha Basheer

**Affiliations:** 1 Department of Chemistry, King Fahd University of Petroleum and Minerals, Dhahran 31261, Saudi Arabia; g201403100@kfupm.edu.sa (M.A.S.); g201310930@kfupm.edu.sa (K.M.A.A.); 2 Interdisciplinary Research Centre for Membranes and Water Security, King Fahd University of Petroleum and Minerals, Dhahran 31261, Saudi Arabia

**Keywords:** electrosynthesis, oxidation, alcohols, undivided cell, droplet assisted, electrocatalyst

## Abstract

This study reports using a droplet flow assisted mechanism to enhance the electrocatalytic oxidation of benzyl alcohol, 2-phenoxyethanol, and hydroxymethylfurfural at room temperature. Cobalt phosphide (CoP) was employed as an active electrocatalyst to promote the oxidation of each of the individual substrates. Surface analysis of the CoP electrocatalyst using scanning electron microscopy (SEM), energy-dispersive X-ray spectroscopy (EDS), X-ray photoelectron spectroscopy (XPS), as well as electrochemical characterization, revealed that it had excellent catalytic activity for each of the substrates studied. The combined droplet flow with the continuous flow electrochemical oxidation approach significantly enhanced the conversion and selectivity of the transformation reactions. The results of this investigation show that at an electrolysis potential of 1.3 V and ambient conditions, both the selectivity and yield of aldehyde from substrate conversion can reach 97.0%.

## 1. Introduction

Electrochemical organic synthesis is one of the most promising organic synthetic tools available, potentially providing a more sustainable and environmentally friendly pathway to produce a wide range of chemical compounds. This is because it eliminates chemical waste by avoiding chemical oxidants and reductants, which is typical of the conventional chemical synthesis approach, and it can operate at mild reaction conditions [1]. More importantly, this synthesis protocol allows for a simple selective transformation of a substrate into useful intermediates or products with high functional group tolerance using only electricity [2]. In this synthesis approach, the applied potential and current to the reaction system can be easily controlled to regulate the redox reactions at the electrode interface [3]. To achieve selective transformation, a “controlled potential” higher than the substrate’s redox potential must be supplied to the electrode to initiate the required chemical reaction.

Carbonyl molecules, such as ketones and aldehydes play key roles in synthetic organic chemistry due to their widespread use as precursors in the pharmaceutical and chemical industries [4]. In the classical chemical oxidation approach, alcohols are often oxidized to aldehydes or ketones in the presence of oxidants such as bromate, chromate, or manganates. However, the hazardous nature of these oxidants, and their likelihood of contaminating the target intermediates, has served as a significant drawback affecting this transformation approach. It has also been challenging to use these oxidants for the oxidation of a wide range of alcohols [5]. The electrochemical oxidation approach was recently advanced as a simple and highly efficient pathway for the selective oxidation of alcohols to primary intermediates. This method offers an appealing alternative to the classical approach. It does not require a chemical oxidant and only generates hydrogen as a by-product of the reaction without product contamination [6].

The published literature has widely explored the direct electrochemical selective oxidation of alcohols to aldehydes or ketones. Most of the documented studies have explored the use of noble metals as catalytic electrodes, such as Ru [6], and Pt [7], in various electrochemical cell configurations, including batch mode [7,8] and continuous flow setup [6]. Furthermore, most of these investigations reported moderate yield and selectivity with extended reaction time for the target aldehydes and ketones. Based on our findings, we believe that combining a microdroplet flow reaction with a continuous flow electrochemical cell should provide a highly efficient and rapid pathway for the selective oxidation of alcohols in the aqueous medium. Over the years, microdroplet flow chemistry has proven to be an important tool in speeding up reaction kinetics and for high throughput applications [6]. These unique characteristics of microdroplet reaction stem from its interfacial features related to its enormous surface-area-to-volume ratio, allowing it to offer a faster reaction rate than the bulk flow counterpart [8,9]. Droplets provide a confined, high-surface-area for chemical reactions, enhancing conversion and selectivity. Droplet-assisted reaction mechanisms are well-known, and several pieces of literature have already been discussed in detail [10,11]. Several reports have attributed this fast reaction rate to be due to the inherent interfacial properties that includes [9,12]: reagent (s) confinement [13,14], partial solvation [9,15,16,17], pH extremes [18,19], and ordered molecule orientation [20,21,22,23,24]. Although most previous research on reaction acceleration with microdroplets is often carried out in organic solvents, aqueous microdroplet reactions are rarely reported.

Over the years, metallic phosphide has evolved as a highly efficient electrocatalyst for oxidation reactions due to its high reactivity in an aqueous medium [25]. These transition metal species facilitate the deployment of oxyhydroxide species in an aqueous medium that has demonstrated excellent catalytic activity and effectiveness in the electrochemical oxidation reaction [26]. Cobalt phosphide (CoP) is highly sought as an active electrocatalyst among these transition metal species due to its ease of preparation. Phosphorus (P) has a lower electronegativity than sulfur, nitrogen, and oxygen [27,28]. It can, however, aid in the formation of oxides, hydroxides, and active phosphate centers on the catalyst surface, resulting in improved electrochemical performance. Even though the electrochemical oxidation active sites are only found on the catalyst’s surface atoms, such reported thick or bulk cobalt phosphide nanostructures suffer from low utilization and exposure of the active sites, limiting their electrochemical activities. As a result, further improvement of the electrochemical oxidation performance of cobalt phosphide is still needed.

Applying the microdroplet flow-continuous flow electrochemical technique, this study investigated the selective oxidation of selected alcohols (benzyl alcohol, 2-phenoxyethanol, and Hydroxymethylfurfural (HMF) for the first time using a modified continuous electrochemical reactor that incorporates a microdroplet flow component. Moreover, CoP on the nickel foam (CoP@NiF) synthesized by the electrodeposition method was employed as the catalytic electrode. Furthermore, several potentials were tuned and optimized to achieve the highest yield and selectivity for the target aldehyde in the shortest period.

## 2. Results and Discussion

The particles of cobalt phosphide were synthesized by an electrodeposition technique using a potentiostat. A three-electrode system was employed, in which the nickel foam serves as a working electrode. The highly conductive Ni foam allows the continuous transfer of electrons across its entire structure, initializing the useful reduction of cobalt (II) ions and dihydrogen phosphate ions to give a net reaction to produce cobalt phosphide [29].

**Figure 1:**
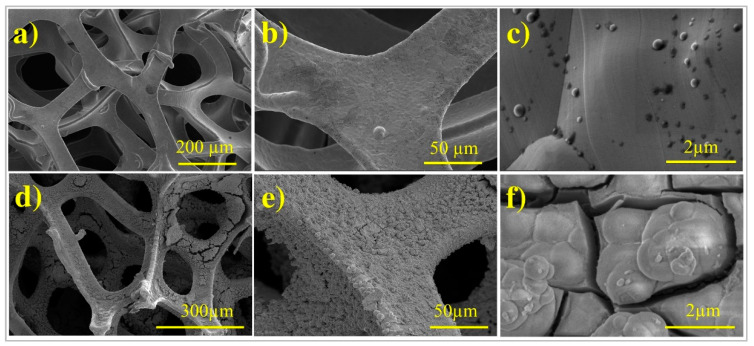
(**a**–**c**) low magnification SEM of bare NiF, (**d**–**f**) Low and high magnification SEM of CoP@NiF.

**Figure 2:**
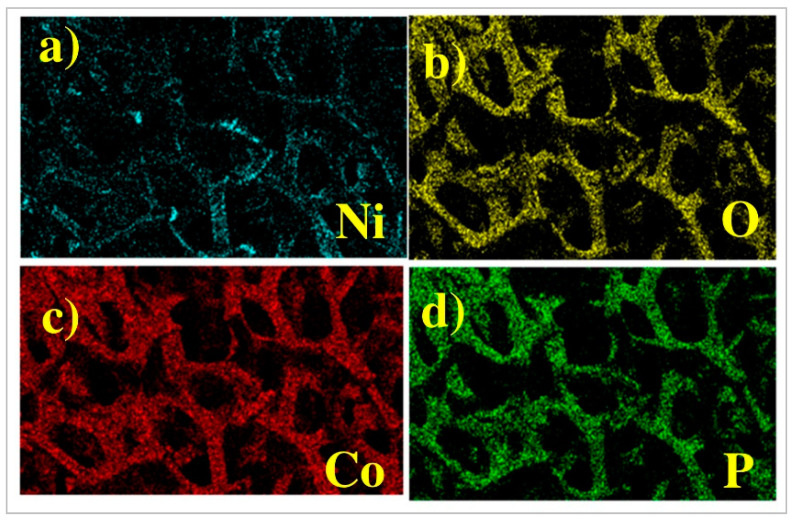
(**a**–**d**) the element mapping of Ni, O, Co, and P, respectively.

The structural details of bare nickel foam (NiF) and modified CoP@NiF were investigated using scanning electron microscopy (SEM). As illustrated (Figure 1a–c), the structure of bare nickel foam was retained while the surface became rougher in the modified CoP (Figure 1d). Low and high magnification of CoP@NiF showed the surface become dense and entirely covered by the CoP particles (Figure 1e,f). The energy-dispersive X-ray spectroscopy (EDS) elemental mapping confirmed the homogenous distribution of Co and P on NiF (Figure 2a–d).

**Figure 3:**
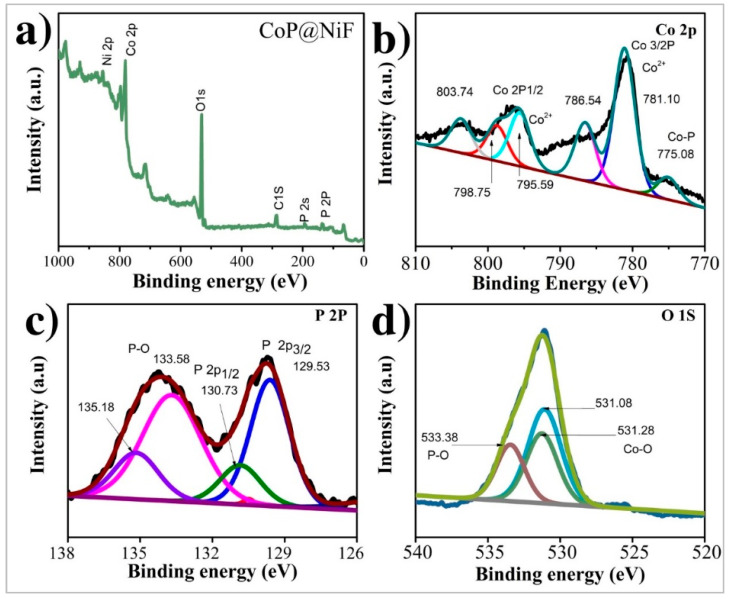
XPS spectra of CoP@NiF. (**a**) survey, (**b**) Co 2P, (**c**) P 2P and (**d**) O 1s.

The surface chemical state of CoP@NiF is investigated using XPS. The survey spectra of CoP@NiF (Figure 3a) show that Co, P, Ni, and O elements coexist in the electrocatalyst. This was completely consistent with the EDX results. The binding energies (BEs) 781.10 (2p_3/2_) and 795.59 (2p_5/2_) eV are ascribed to the binding energies Co 2p_3/2_ and Co 2p_1/2_ in CoP, respectively whereas the BEs 779.6 and 795.1 eV are attributed to (2p_3/2_) and (2p_5/2_) [30]. Satellite peaks are the two remaining deconvoluted peaks, found at 786.54 eV and 803.74 eV. Furthermore, the high-resolution XPS spectra of P 2p core levels can be deconvolved into three peaks, respectively positioned at 129.5 eV for P 2p_3/2_, 130.7 eV for P 2p_1/2_, and 133.58 eV for oxidized P species. The phosphate is represented by a wide peak at 135.4 eV, indicating surface oxidation [31,32]. The peaks at 781.2 and 134.4 eV are attributed to oxidized Co and P species formed by the surface oxidation of CoP. It is proposed that the peaks at 780.4 eV and 133.5 eV develop as a result of the unavoidable interaction between the CoP surface and oxygen. The high-resolution C spectra of O 1s supports the findings, which can be deconvoluted into three peaks of P-O (533.2 eV), C-O (531.9 eV), and Co-O (531.3 eV).

Furthermore, compared to the binding energies of metallic Co 2p_3/2_ (778.1–778.2 eV) and elemental P 2p_3/2_ (130.2 eV), the peaks of Co 2p_3/2_ and P 2p_3/2_ have shifted positively and negatively. Revealing electron transfer from Co atoms to P atoms in CoP sheets, suggesting a partial positive charge for Co centers and a partial negative charge for P centers [33]. This type of charge transfer facilitates the adsorption and desorption of intermediates and products during electrochemical oxidation of various substrates [34,35].

**Figure 4:**
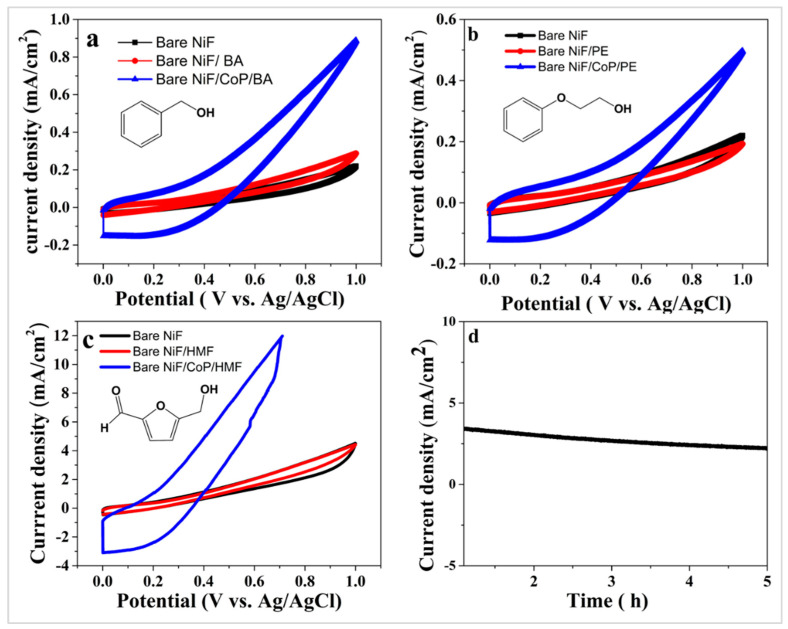
The CV of a different substrate of CoP@NiF electrocatalysts in 1.0 M KOH: (**a**) benzyl alcohol, (**b**) 2-phenoxyethanol, (**c**) HMF, (**d**) current-time curve of the CoP@NiF.

Cyclic Voltammetry (CV) studies were performed to examine the prepared electrode’s catalytic activity for alcohol oxidation. For this approach, a three-electrode system cell was used in which bare nickel foam, platinum electrode, and Ag/AgCl serve as the working, counter, and reference electrodes, respectively. The CV results indicate that after adding benzyl alcohol to the acetonitrile-water mixture solvent, the current density was increased significantly, which was attributed to the alcohol-oxidation reaction (Figure 4a). The same was observed with the addition of 2-phenoxyethanol in Figure 3b. However, 2-phenoxyethanol shows a lower current density increment compared to benzyl alcohol.

Furthermore, the addition of HMF also enhanced the current density. These findings show that the prepared CoP electrocatalyst exhibits significant catalytic activity towards the oxidation of alcohols. It is worth mentioning that the bare nickel foam showed lower current density and low capacitive current for all the substrates, as illustrated in Figure 4. Figure 3d depicts a five-hour duration chronoamperometry curve for the CoP@NiF electrode in the presence of the electrolytes. No significant changes in current density were observed for the entire period investigated, demonstrating the chemical stability of the electrocatalyst under the applied experimental conditions.

Our investigation of the reaction condition starts with benzyl alcohol (1a), which was initially exposed to a batch condition (no droplet flow) in an undivided cell (Table 1). This approach yielded a 47% selectivity for benzaldehyde (1b) after 1 h of electrolysis at 2.0 V. A mixture of acetonitrile and water (1:1) was employed as a solvent to increase the starting material’s solubility. The selectivity of generating benzaldehyde was achieved by employing a low concentration of potassium hydroxide, which reduced the possibility of the Cannizaro reaction.

The continuous electrochemical flow cell with a droplet flow mechanism was then used in the next phase. The selectivity of generating benzaldehyde with this method reached 94% for the same experimental conditions, whereas the (1a) selectivity was only 47.0%. The increased conversion and product selectivity facilitated by the flow droplet mechanism was attributed to the enhanced surface reaction at the electrode interface. The effect of electrical potential variation on the conversion and selectively was also studied, as shown in Table 1. Although there was no change in the conversion of benzyl alcohol at lower potentials (0.5–1.5 V), the potential was discovered to impact the product selectivity significantly.

In the next stage, we investigated the transformation of 2-phenoxyethanol using the droplet flow-assisted electrochemical oxidation approach. According to reports, at a potential of 1.6 V, the oxygen atom of a phenoxy molecule of 2-phenoxyethanol may be oxidized to generate a positively charged radical that can then be utilized to construct polymer chains. Its oxidation potential is approximately 1.0 V higher than that of most phenols. Based on these findings, we limit our voltage variation in this study to 1.6 V for 2-phenoxyethanol conversion. The experimental results are displayed in Table 2. In entry 1, a mixture of CH_3_CN/H_2_O (1:1) was used; only a 6.0% conversion was achieved. This lower conversion may be due to the insolubility of the substrate, so no electrochemical oxidation is expected to occur at this condition. Entry 2 involves a control experiment with the bare nickel foam without the electrocatalyst, which also shows a lower conversion of 8% for 2-phenoxyethanol and a selectivity of 12%. In the case of entry 3, when NaOH 1.0 M was used as a supporting electrolyte, 56.5% conversion of 2-phenoxyethanol was achieved (entry 3). In the former entry 4, the major products of this entry were phenol and formic acid with 92%. According to the HPLC results, the major products of this reaction were phenol and formic acid. It is essential to keep in mind that the formation of formic acid increased over time, reaching its peak at 40 min of electrolysis and then immediately disappearing after 1.0 h.

Next, the conversion of HMF with droplet flow-assisted electrochemical oxidation was examined in an alkaline medium (1.0 M NaOH) in the presence of 20 mM of HMF. Similar to the previous transformation experiment, the electrical potential was varied from 0.5 to 1.6 V. Table 3 shows the conversion of HMF and the selectivity of the formed 2,5-furandicarboxylic acid (FDCA) using the droplet flow electrochemical oxidation approach. As shown, the increase in potential from 0.5 and 0.7 V led to an increase in conversion and selectivity (entries 1 and 2). In contrast, entry 3 showed that the selectivity decreased as the voltage increased to 1.0 V. Furthermore, high selectivity and high conversion were noted at high voltage, as shown in entries 4 and 5. It is worth mentioning that at potentials greater than 1.3 V, a slight decrease in the selectivity was observed, which could be attributed to product decomposition at the higher voltage. The optimum condition was 1.3 V for 1.0 h to get the high selectivity for FDCA, which was attributed to the enhancement from the droplet-assisted method. In comparison, a bath mode was applied, and the reaction took 2.0 h to achieve the same conversion and selectivity.

With the insight that CoP@NiF supported catalysts can drive the oxidation of various alcohols, the oxidation of BA, 2-PE, and HMF was investigated at room temperature and atmospheric pressure. Figure 4 depicts the decrease in alcohol concentration and the formation of aldehyde as a function of reaction time. It is clear that the formation of benzaldehyde occurred even within the first 10 min. The concentration of benzaldehyde increased dramatically as the reaction progressed. It is worth noting that no significant benzoic acid was detected in the reaction medium, indicating that the electrode has a high selectivity to produce only benzaldehyde. However, as shown in Figure 4b, the change in 2-PE concentration over time revealed two main products: phenol and acetic acid.

On the other hand, HMF oxidation produced intermediate and interstitially high yields of FDCA in a short time. The reaction had reached 60% FDCA formation after 30 min. The fast conversion indicates that the main difference between microdroplets and bulk solutions is the large surface area and confined space of the droplet on all different substrates [24].

**Figure 5:**
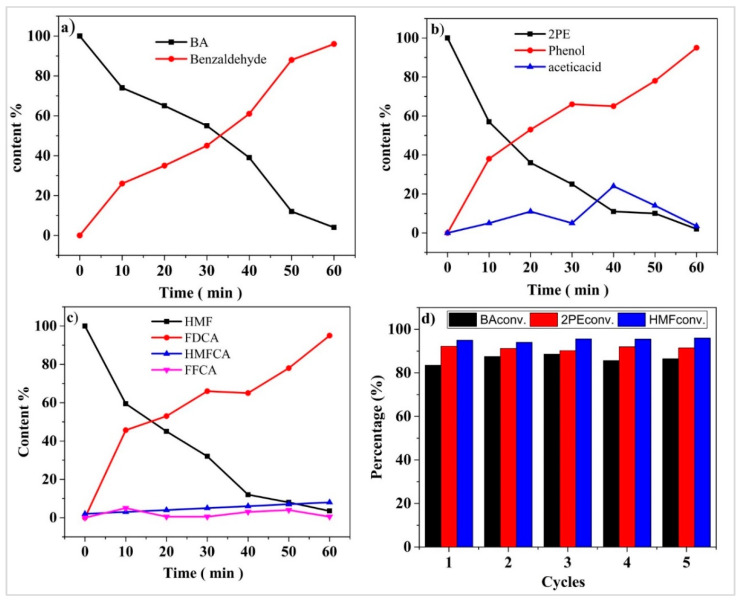
Evolution of reactant substrate and product concentrations for (**a**) BA, (**b**) 2-PE, and (**c**) HMF. (**d**) Effect of recycling on the electrocatalytic oxidation of CoP@NiF for the conversion of different substrates.

The ability to recycle an electrode is a critical factor in determining electrode stability. The recyclability of the CoP@NiF electrode was investigated by electrolyzing it several times. As shown, the CoP@NiF is an efficient, recyclable electrocatalyst for the selective electrochemical oxidation of BA, 2-PE, and HMF substrates. The electrochemical oxidation of the substrates as a function of different runs is illustrated in Figure 5d. Table 4 compares our present work to previous work in the literature, and it demonstrates fast and high conversion that is superior to the published literature.

## 3. Materials and Methods

### 3.1. Chemicals

Cobalt (II) chloride hexahydrate (CoCl_2_·6H_2_O), sodium dihydrogen phosphate (NaH_2_PO_4_), potassium chloride, nickel foam, benzyl alcohol (BA), 2-phenoxyethanol (2-PE), hydroxymethylfurfural (HMF), potassium hydroxide (KOH), ammonium formate, tetrabutylammonium tetrafluoroborate (Bu_4_NBF_4_), and acetonitrile. All reagents were purchased from Sigma Aldrich and used without any further purification.

### 3.2. Synthesis of CoP@Ni Foam

**Scheme 1:**
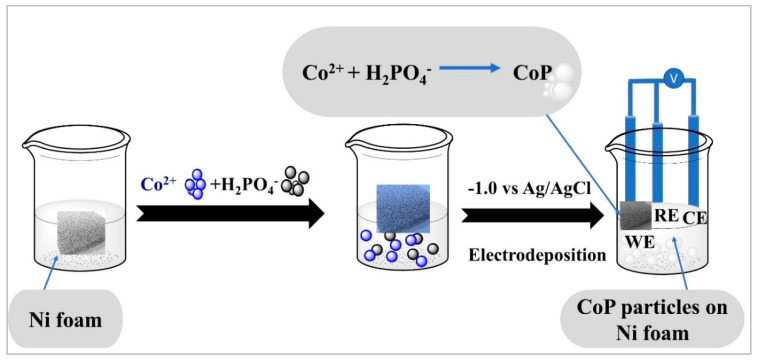
Schematic diagram of CoP@NiF synthesis.

The CoP was deposited on the nickel foam by using the electrodeposition method. In brief, cobalt chloride hexahydrate (CoCl_2_·6H_2_O) and sodium dihydrogen phosphate (NaH_2_PO_4_) were used as cobalt and phosphorus sources, respectively. A supporting electrolyte of KCl was dissolved in a specific volume of deionized water under nitrogen gas purging. The electrodeposition of CoP film was performed by using a standard three-electrode system: the working electrode was cleaned bare NiF, the counter electrode was platinum, and the reference electrode was silver/silver. Then, the applied potential of −1.0 V was polarized into the Ni foam surface. Finally, the working electrode was rinsed with deionized water and dried at room temperature after the electrodeposition. The method of synthesis is shown in Scheme 1.

### 3.3. Structural Characterization

The surface characterization of the prepared CoP@NiF electrode was performed using field emission scan electron microscopy (FE-SEM) coupled with energy-dispersive X-ray spectroscopy (EDS). An X-ray photoelectron spectrometer was used to collect the surface elemental composition (Thermo Scientific ESCALAB 250Xi, Waltham, MA, USA). The C1s hydrocarbon peak at 284.60 eV was used to calibrate all binding energies.

### 3.4. Droplet-Assisted Electrochemical Oxidation of Alcohols

**Scheme 2:**
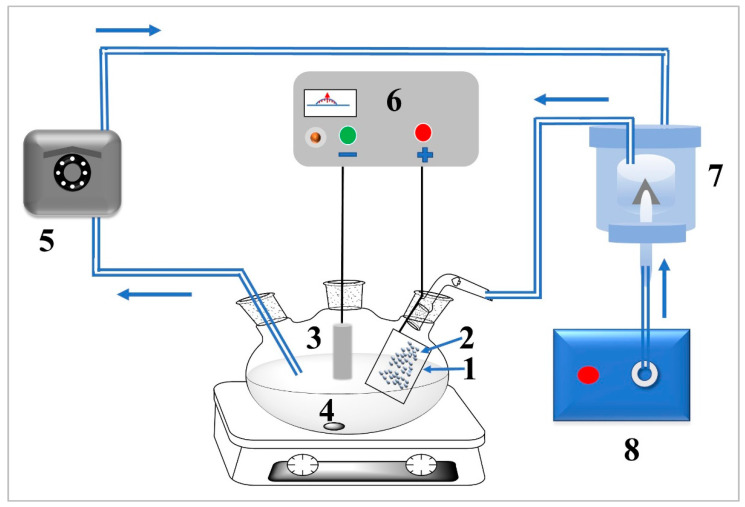
The experimental setup of the droplet assisted electrosynthesis reactor includes: (1) CoP@NiF (the anode), (2) droplet formation, (3) platinum electrode acting as the cathode, (4) magnetic stirrer, (5) peristaltic pump, (6) DC power supply, (7) nebulizer junction for mixing natural air with the untreated sample to form a droplet spray at the anode surface, and (8) air pump.

All electrochemical experiments were carried out using CH Instruments 1140A potentiostats (CH Instruments, Inc., Austin, TX, USA). An undivided electrochemical cell (EuroCell) with a 50 mL volume (working solution, 0.21 L and GSG-ME) was modified to suit a droplet-flow assisted configuration. Scheme 2 shows the schematic diagram of the droplet-assisted electrochemical flow reactor used in this work. A nickel foam was used as the anode, while the cathode was a platinum electrode and was connected to a direct current power source (Ealing High voltage power supply, 0–50 V). The electrodes were placed 1.5 cm from the reactor’s bottom and 2.0 cm apart as an inter-electrode gap. An air pump connected to the sample flow recirculatory system was used to create a nebulized mist of the sample solution directed at the anode surface.

For all the experiments, the bulk sample solutions were stirred magnetically. The electrochemical droplet assisted condition setup was set CoPNiF as the anode, and the Pt electrode was used as the cathode. A mixture of 30 mL of acetonitrile and H_2_O (1:1) as supporting electrode and 0.2 mmol of Bu_4_NBF_4_. The concentration of the different alcohol substrates was 2.0 mmol. All the experimental electrolysis was repeated three times.

### 3.5. Liquid Chromatography Analysis

High-performance liquid chromatography (HPLC, Shimadzu) was used in monitoring the electrochemical oxidation process. Data acquisition and quantification are conducted with Lab Solutions, LC Ver.5.91, Shimadzu. An autosampler-CTC-Pal (Analytics, NC, USA) ultra IBD column (100 × 2.1 mm × 3 μm particle size; Restek, PA, USA) was used for the separation of target analytes. Isocratic elution using solvent A (5.0 mM ammonium formate) and solvent B (acetonitrile) was applied at a flow rate of 0.04 mL/min. The injection volume was 2.0 µL with the isobaric condition.

Standard solutions of reactants and products, such as benzyl alcohol, 2-phenoxyethanol, and HMF, were used to obtain the calibration curves. The alcohol concentrations and their oxidation products were determined from the peak areas. Percent (%) conversion and selectivity were calculated using the following formulas:

(1) $$\mathrm{Conversion}\;\%=\frac{\left(C_{r_{0}}-C_{r}\right)}{C_{r_{0}}}*100$$

(2) $$\mathrm{Selectivity}=\frac{C_{p}}{\left(C_{r_{0}}-C_{r}\right)}*100$$

$C_{r_{0}}$ denotes the reactant’s initial concentration, C_r_ and C_p_ denote reactant’s and product’s concentration during the reaction, respectively [40].

## 4. Conclusions

To conclude, a fast and straightforward transformation of alcohols and HMF into the corresponding intermediates using droplet assisted flow with a continuous flow electrochemical reactor was demonstrated for the first time. The selectivity and yield of benzaldehyde can be tuned by electrolysis potential. The results show that at 1.5 V and 318.15 K, both the selectivity and the yield of benzaldehyde can reach above 98.8% with the system under ambient conditions. An accelerated droplet synthesis approach of alcohol and its derivatives has been achieved.

## Figures and Tables

**Table 1:** Optimization of droplet-assisted electrochemical oxidation of benzyl alcohol *.

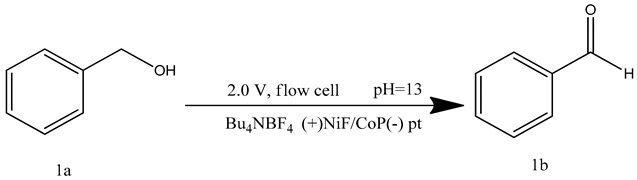			
Entry	Potential (V)	%Conversion	% Selectivity
Droplet assisted method	0.5	45.2	54.0
	1.0	45.2	78.4
	1.5	46.1	82.5
	2.0	83.5	94.0
Without droplet	2.0	32.0	47.0

* Reaction conditions: Benzyl alcohol (2.0 mmol) and KOH (0.01 M), the temperature at 25 °C, electrolysis time is 60 min. The stirring rate is 250 rpm, and the flow rate is 12.5 µL/min.

**Table 2:** Optimization of droplet-assisted electrochemical oxidation of 2-phenoxyethanol *.

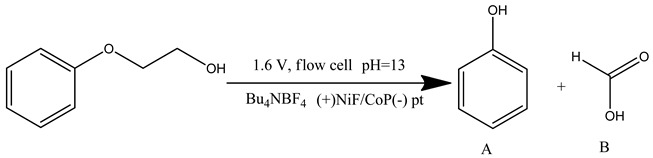			
Entry	Potential (V)	% Conversion	% Selectivity
1	0.5	6.0	10.0
2	1.0	8.0	12.0
3	1.2	56.5	71.0
4	1.6	92.0	96.0

* Reaction conditions: Phenoxyethanol (2.0 mmol), NaOH (1.0 M) [b] determined by HPLC, temperature at 25 °C, electrolysis time is 60 min. The stirring rate is 250 rpm, and the flow rate is 12.5 µL/min.

**Table 3:** Optimization of droplet assisted electrochemical oxidation of HMF *.

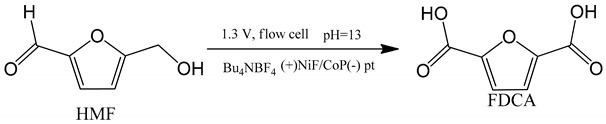			
Entry	Potential (V)	% Conversion	% Selectivity
1	0.5	66.0	68.5
2	0.7	88.0	91.6
3	1.0	89.2	92.0
4	1.3	95.0	96.0
5	1.6	86.0	88.0

* Reaction conditions: HMF (20 mM) and KOH (1.0 M), the volume of electrolyte (30 mL), 5 mL of HMF in the nebulizer box, and temperature at 25 °C for 60 min. The stirring rate is 250 rpm, and the flow rate is 12.5 µL/min.

**Table 4:** Comparison of electrochemical oxidation of alcohol using various electrocatalyst.

Catalyst	Substrate	Product	Conv. %	Yield %	Condition	Time (min)	Ref.
Nickel oxy-hydroxide electrode	BA (*)	Benzaldehyde, Benzoic acid	-	86%	0.01 M NaOH	300	[36]
Au NP- modified carbon-fiber-thread electrode	BA	Benzaldehyde	-	50–55	KOH, LiClO_4_ [a]	-	[7]
	1-PE	Acetophenone	-	38–65	KOH, LiClO_4_		
TEMPO (0.1 mmol)	BA	Benzaldehyde	92.0	-	Carbonate buffer	120	[37]
NHPI (0.1 mmol)	BA	Benzaldehyde	57.0		Acetate buffer		
Co-P on Cu foam	HMF	FDCA	99.0	85.3	Deep eutectic solvents, 0.5 M NaHCO_3_	-	[38]
Carbon anode	BA	Benzaldehyde	-	95.0	Undivided, 30–34 °C HCl as supporting electrolyte nitrate as mediator	-	[39]
CoP@NiF	BA	Benzoic acid	95.0	96.0	Droplet assisted method	60	This work
	2-PE	Phenol & CH_3_CO_2_H	96.0	96.0			
	HMF	FDCA	95.0	96.0			

(*) BA, benzyl alcohol; TEMPO: (2,2,6,6-tetrame- thylpiperidin-1-yl)oxyl, (NHPI) N-hydroxyphthalimide, 2-PE (2-phenoxyethanol).

## Data Availability

Data is contained within the article.
